# Exploring d-xylose oxidation in *Saccharomyces cerevisiae* through the Weimberg pathway

**DOI:** 10.1186/s13568-018-0564-9

**Published:** 2018-03-05

**Authors:** Lisa Wasserstrom, Diogo Portugal-Nunes, Henrik Almqvist, Anders G. Sandström, Gunnar Lidén, Marie F. Gorwa-Grauslund

**Affiliations:** 1 Division of Applied Microbiology, Department of Chemistry, Lund University, PO Box 124, 221 00 Lund, Sweden; 2 Department of Chemical Engineering, Lund University, PO Box 124, 221 00 Lund, Sweden; 3 Present Address: Harboes Bryggeri A/S, Spegerborgvej 34, 4230 Skælskør, Denmark; 4 Present Address: Novozymes A/S, Krogshøjvej 36, 2880 Bagsværd, Denmark

**Keywords:** d-Xylose, Weimberg pathway, *Saccharomyces cerevisiae*, d-Xylonate dehydratase, *Caulobacter crescentus*, Iron–sulfur clusters

## Abstract

Engineering of the yeast *Saccharomyces cerevisiae* towards efficient d-xylose assimilation has been a major focus over the last decades since d-xylose is the second most abundant sugar in nature, and its conversion into products could significantly improve process economy in biomass-based processes. Up to now, two different metabolic routes have been introduced via genetic engineering, consisting of either the isomerization or the oxido-reduction of d-xylose to d-xylulose that is further connected to the pentose phosphate pathway and glycolysis. In the present study, cytosolic d-xylose oxidation was investigated instead, through the introduction of the Weimberg pathway from *Caulobacter crescentus* in *S. cerevisiae*. This pathway consists of five reaction steps that connect d-xylose to the TCA cycle intermediate α-ketoglutarate. The corresponding genes could be expressed in *S. cerevisiae*, but no growth was observed on d-xylose indicating that not all the enzymes were functionally active. The accumulation of the Weimberg intermediate d-xylonate suggested that the dehydration step(s) might be limiting, blocking further conversion into α-ketoglutarate. Although four alternative dehydratases both of bacterial and archaeon origins were evaluated, d-xylonate accumulation still occurred. A better understanding of the mechanisms associated with the activity of dehydratases, both at a bacterial and yeast level, appears essential to obtain a fully functional Weimberg pathway in *S. cerevisiae*.

**Electronic supplementary material:**

The online version of this article (10.1186/s13568-018-0564-9) contains supplementary material, which is available to authorized users.

## Introduction

**Fig. 1:**
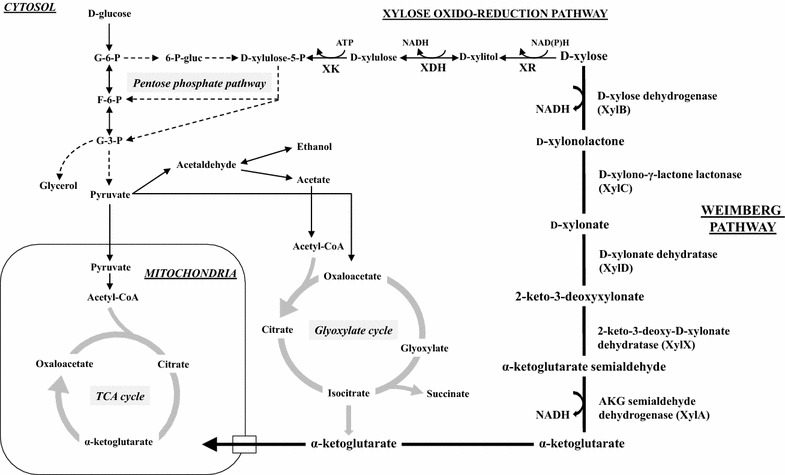
A condensed metabolic map describing d-xylose oxidation via the Weimberg pathway. Five enzymatic reactions convert d-xylose to α-ketoglutarate that enter the central cellular metabolism via the TCA cycle. For comparison, the oxido-reduction of d-xylose to d-xylulose through the oxido-reduction pathway (XR/XDH) is also shown. Required cofactors (ATP, NADH or NADPH) are indicated for the two xylose pathways while the central cellular metabolism is presented in a simplified scheme. *XR* xylose reductase, *XDH* xylitol dehydrogenase, *XK* xylulose kinase, *TCA cycle* tricarboxylic acid cycle

The pentose sugar d-xylose is an abundant carbon source in nature. It is a dominating part of hemicellulose—in the form of xylan—in hardwoods and agricultural crops, but is also present to a lower extent in softwoods. Several microorganisms are capable of metabolizing d-xylose through isomerization, oxido–reduction or oxidation reactions. Most bacteria, such as *Escherichia coli*, use the d-xylose isomerization pathway, in which d-xylose is converted to d-xylulose by d-xylose isomerase (XI) (Jeffries 1983). In contrast, the oxido–reduction route, involving a two-step process catalyzed by d-xylose reductase (XR) and xylitol dehydrogenase (XDH), is more common in yeasts such as *Scheffersomyces stipitis* (Du Preez and Prior 1985). In both routes, xylulose kinase (XK) converts d-xylulose into d-xylulose-5-P that can be further metabolized via the pentose phosphate pathway (PPP) and glycolysis (Wang and Schneider 1980). The Weimberg pathway, first described in the bacterium *Pseudomonas fragi* in 1960s, is a different metabolic route based on d-xylose oxidation (Weimberg 1961). This route that has received increasing attention in the past decade, has been characterized in several prokaryotes, including the freshwater bacterium *Caulobacter crescentus* and the halophilic archaeon *Haloferax volcanii* (Johnsen et al. 2009; Stephens et al. 2007). In this metabolic pathway, d-xylose is oxidized to the tricarboxylic acid (TCA) cycle intermediate α-ketoglutarate in five enzymatic steps, without carbon loss. d-Xylose is initially oxidized to d-xylono-γ-lactone by a d-xylose dehydrogenase (XylB, encoded by *xylB*), that is further converted to the intermediate d-xylonate by a d-xylono-γ-lactone lactonase (XylC, encoded by *xylC*). d-Xylonate is then subjected to two dehydration reactions by a d-xylonate dehydratase (XylD, encoded by *xylD*) and a 2-keto-3-deoxy-d-xylonate dehydratase (XylX, encoded by *xylX*), yielding 2-keto-3-deoxy-d-xylonate and then α-ketoglutarate semialdehyde. In the final step, the semialdehyde is oxidized by α-ketoglutarate semialdehyde dehydrogenase (XylA, encoded by *xylA*) into α-ketoglutarate. The entry point of carbon into the central cellular metabolism is thus completely different from both the XI and the XR-XDH pathways (Fig. 1). Heterologous expression of the Weimberg pathway from *C. crescentus* has enabled bacteria such as *Pseudomonas putida* and *Corynebacterium glutamicum* to grow on d-xylose as the sole carbon source (Meijnen et al. 2009; Radek et al. 2014). However, to the best of our knowledge, the complete Weimberg pathway has not yet been established in fungal species.

*Saccharomyces cerevisiae* is an extensively investigated yeast due to its unique features and potential to be applied in biotechnological processes aiming for the production of a large range of chemical compounds. Among others, the production of biofuels (e.g. ethanol, farnesene, isobutanol), pharmaceutical drugs (e.g. artemisinic acid) and carboxylic acids has received large attention from both academia and industry (Abbott et al. 2009; Borodina and Nielsen 2014; Buijs et al. 2013; Paddon et al. 2013). Since *S. cerevisiae* is not capable of naturally fermenting d-xylose, considerable research efforts have been invested in the engineering and optimization of d-xylose assimilation pathways based on XR-XDH or XI in *S. cerevisiae*, in particular for the production of ethanol or acetyl-CoA derived products [see reviews by e.g. (Hahn-Hägerdal et al. 2007; Kim et al. 2013; Van Maris et al. 2007)]. In that context, the Weimberg pathway that converts d-xylose to α-ketoglutarate that enters the metabolism via the TCA cycle could be relevant for the production of carboxylic acids produced or derived from the TCA-cycle. Potential applications for α-ketoglutarate are as a dietary supplement, in medical products, and as a platform chemical for the synthesis of heterocyclic compounds (Otto et al. 2011). Importantly, α-ketoglutarate can be used as a precursor for the production of acids with novel characteristics that are not yet being produced on a large scale, such as glutaric, glutaconic and glutamic acids (Bajaj and Singhal 2011; Djurdjevic et al. 2011; Dutta et al. 2013; Fink 2013; Sailakshmi et al. 2013; Sung et al. 2005). Chemical synthesis of α-ketoglutarate is possible through various routes, but problematic since it is a multi-step reaction partly involving toxic chemicals (Otto et al. 2011). Therefore, more attention is being directed to the biotechnological production of α-ketoglutarate since this constitutes an environmentally friendly alternative (Aurich et al. 2012; Otto et al. 2011).

The upper part of the Weimberg pathway has already been successfully introduced in *S. cerevisiae* (Salusjärvi et al. 2017; Toivari et al. 2012). However, biomass formation through the Weimberg pathway can only occur if d-xylose is fully oxidized to α-ketoglutarate.

In the present work, genes from the complete Weimberg pathway from *C. crescentus* were introduced into *S. cerevisiae* via genetic engineering to evaluate the possibility to oxidise d-xylose, with the long-term aim to produce α-ketoglutarate-derived bulk chemicals of industrial interest. The impact of replacing the d-xylonate dehydratase from *C. crescentus* (*Cc* XylD) by homologous enzymes of bacterial and archaeal origin on the yeast physiology and the functionality of the Weimberg pathway was also assessed as it has been shown that the dehydration reaction of d-xylonate to 2-keto-3-deoxy-d-xylonate catalyzed by XylD in *C. crescentus* is dependent on the coordination of Fe–S cluster(s) to three conserved cysteine residues (Andberg et al. 2016).

## Materials and methods

### Strains, media and culture conditions

*Escherichia coli* strain NEB5-α (New England Biolabs) was used for sub-cloning of plasmid DNA and grown at 37 °C in Luria–Bertani (LB) broth containing 5 g L^−1^ yeast extract, 10 g L^−1^ peptone, 5 g L^−1^ NaCl, pH 7.5. Bacterial transformants were selected on LB agar plates (15 g L^−1^ agar) supplemented with 50 mg L^−1^ ampicillin for 16 h at 37 °C. Yeast strains were grown at 30 °C and 180 rpm in an orbital shaker under non-selective conditions in Yeast Peptone Dextrose (YPD) medium containing 10 g L^−1^ yeast extract, 20 g L^−1^ peptone and 20 g L^−1^ d-glucose. Competent yeast cells were prepared and transformed according to the high efficiency LiAc protocol but DMSO was added before heat shock at 42 °C for 20 min (Gietz and Schiestl 2007). Transformants generated using the CRISPR–Cas9 system (Stovicek et al. 2015) were selected on YPD media supplemented with 200 mg L^−1^ geneticin and 100 mg L^−1^ nourseothricin (Jena Bioscience, Germany) to allow selection of the Cas9-kanMX and gRNA-natMX plasmids. When amdSYM was used as the selection marker, transformants were plated on synthetic media containing acetamide as the sole nitrogen source (SM-Ac) by following the protocol described by (Solis-Escalante et al. 2013). To select for cells that had recycled the amdSYM marker, cells were plated on SM containing 2.3 g L^−1^ fluoroacetamide (SM-FAc). Genomic *S. cerevisiae* DNA to be used for PCR amplification was extracted using the LiOAc/SDS lysis protocol (Lõoke et al. 2011). All restriction enzymes used where Fastdigest enzymes purchased from ThermoFisher Scientific (Massachusetts, USA). All chemicals and sugars were purchased from Sigma Aldrich (Missouri, USA).

### Construction of expression cassettes, targeting fragments and plasmids

The plasmids used in this study are listed in Table 1. Primers are listed in Additional file 1: Table S1. The Weimberg pathway from *C. crescentus* was codon-optimized for *S. cerevisiae* and designed with promoter–terminator pairs already included, and ordered from GeneScript USA Inc. (New Jersey, USA). The five *C. crescentus* genes were delivered on four pUC57 vectors, pUC57-*xylBC*, pUC57-*xylD*, pUC57-*xylX*, and pUC57-*xylA*. The coding regions for *xylB*, *xylC*, *xylD*, *xylX* and *xylA* were inserted in between the strong constitutive promoter–terminator pairs of the genes *TEF1*, *TPI1*, *GPM1*, *GPD* and *FBA1*, respectively, known to be active on d-xylose (Jin et al. 2004). pUG6-amdSYM2 harboring the two infrequent endonuclease sites *Asi*SI and *Asc*I, was constructed to form a platform for long pathway fragments by amplifying a fragment with primers MCS_Cassette_f and _r using pUG6-amdSYM as template (Solis-Escalante et al. 2013). The generated fragment was digested with *Sac*I and *Sal*I, and AGE-purified (agarose gel electrophoresis). The template pUG6-amdSYM was digested with *Sac*I, *Sal*I and *Bbs*I and simultaneously dephosphorylated with FastAP, followed by AGE-purification. The digested fragment and vector backbone was ligated, generating pUG6-amdSYM2, which was fully validated by sequencing.

**Table 1:** Plasmids used in the present study

Plasmid name	Relevant genotype	References
pUG-amdSYM	(*A.g.*)*TEF1p*-*amdS*-*(A.g.)TEFt*	Solis-Escalante et al. (2013)
pUG-amdSYM2	*Asc*I-MCS;(*A.g.*)*TEF1p*-*amdS*-*(A.g.)TEFt*; *Asi*SI	This study
pUC57-xylBC	*TEF1p*-*xylB*-*TEF1t*-*TPI1p*-*xylC*-*TPI1t*	This study
pUC57-xylD	*GPM1p*-*xylD*-*GPM1t*	This study
pUC57-xylX	*GPDp*-*xylX*-*GPDt*	This study
pUC57-xylA	*FBA1p*-*xylA*-*FBA1t*	This study
pUC57-xad_Hv	*GPM1p*-*xad_Hv*-*GPM1t*	This study
pUC57-xad_Bc	*GPM1p*-*xylD_Bc*-*GPM1t*	This study
pUC57-yjhG_Ec	*GPM1p*-*yjhG_Ec*-*GPM1t*	This study
pUC57-xylD_El	*GPM1p*-*xylD_El*-*GPM1t*	This study
Cas9	*TEF1p-Cas9-CYC1t*_kanMX	Stovicek et al. (2015)
gRNA.ADE2	*SNR52p-gRNA.ADE2-SUP4t*_natMX	Stovicek et al. (2015)
pLWA25	*SNR52p-gRNA.GRE3-SUP4t*_natMX	This study
pLWA36	*SNR52p-gRNA.xylD-SUP4t*_natMX	This study
pAGS7	pUG-amdSYM2-*TEF1p*-*xylB*-*TEF1t*-*TPI1p*-*xylC*-*TPI1t*	This study
pAGS8	pUG-amdSYM2-*GPM1p*-*xylD*-*GPM1t*-*GPDp*-*xylX*-*GPDt*-*FBA1p*-*xylA*-*FBA1t*	This study
pAGS8H	pUG-amdSYM2-*GPM1p*-*xad_Hv*-*GPM1t*-*GPDp*-*xylX*-*GPDt*-*FBA1p*-*xylA*-*FBA1t*	This study
pAGS8B	pUG-amdSYM2-*TEF1p*-*xylB*-*TEF1t*-*GPM1p*-*xylD*-*GPM1t*-*GPDp*-*xylX*-*GPDt*-*FBA1p*-*xylA*-*FBA1t*	This study
pAGS8HB	pUG-amdSYM2-*TEF1p*-*xylB*-*TEF1t*-*GPM1p*-*xad_Hv*-*GPM1t*-*GPDp*-*xylX*-*GPDt*-*FBA1p*-*xylA*-*FBA1t*	This study

pAGS7 was generated by digesting the template pUG6-amdSYM2 with *Asc*I and *Kas*I simultaneously dephosphorylated with FastAP, followed by AGE-purification. pUC57-*xylBC* was digested with *Asc*I, *Kas*I and *BspH*I, and AGE-purified. The resulting fragments and templates were successfully ligated, generating pAGS7, which was validated by restriction fragment analysis using *Nsb*I. pAGS8 was generated by digesting the template pUG6-amdSYM2 with *Sma*I followed by AGE-purification. The pUC57-*xylD* was digested with *Rru*I, *Fsp*I and *Afe*I; pUC57-*xylX* was digested with *Sma*I, *Fsp*I and *Afe*I; pUC57-*xylA* was digested with *Sma*I, *Fsp*I, and all the digested fragments were AGE-purified. The resulting fragments and template were assembled using In-Fusion HD Enzyme Mix (Clontech Laboratories, California, USA) and the resulting plasmid was validated. pAGS8H was constructed by digesting the template pAGS8 with *Sal*I and dephosphorylated with FastAP, followed by AGE-purification. pUC57-*xad_Hv* was digested with *Sal*I, and the resulting, purified fragments were ligated. The generated vector was validated and correct orientation was verified with PCR using primers xad_Hv_f/GPM1t_r and restriction fragment analysis using *Nde*I. pAGS8B and pAGS8HB were generated by cloning the *TEFp*-*xylB*-*TEFt* from AGS7 into pAGS8 and pAGS8H, respectively. pAGS7 was digested with *Asc*I and *Afe*I and pAGS8 and pAGS8H were digested with *Asc*I and *Sma*I, and the resulting, purified fragments were ligated. The obtained vectors pAGS8B and pAGS8HB were validated by PCR and restriction fragment analysis using *Nde*I and *Ale*I.

Three homologs to *C. crescentus xylD* were selected, codon-optimized for *S. cerevisiae* using GeneArt (ThermoFisher Scientific, Massachusetts, USA) and ordered from GeneScript USA Inc. (New Jersey, USA). The gene sequences were retrieved from the Integrative Microbial Genomes database supplied by the Joint Genome Institute (Markowitz et al. 2014).

The CRISPR–Cas9 system developed by Stovicek et al. (2015) was used for selective targeting of sites. The gRNA target sequence for *GRE3* was selected using the CRISPy tool (Ronda et al. 2014) and for *xylD* using the E-CRISP tool (Heigwer et al. 2014). The gRNA plasmids pLWA25 and pLWA36 were constructed by amplifying the gRNA backbone as described by Stovicek et al. (2015) using a phosphorylated forward primer, 102 for pLWA25 and 263 for pLWA36, containing a tail with the specific 20-bp target sequence for *GRE3* or *xylD*, respectively, together with the phosphorylated reverse primer 103. As a template the two-micron-based replicative gRNA plasmid for targeting *ADE2* containing the natMX dominant marker for selection was used.

Targeting fragments for nested double homologous integration of pAGS7, pAGS8B and pAGS8HB into the *GRE3* locus and pAGS8 into the intergenic region between *VAC17* and *MRC1* were constructed by PCR amplification. About 400 bp of the 5′ upstream region and the 3′ downstream region of *GRE3* were amplified from genomic *S. cerevisiae* CEN.PK 113-7D DNA using Phusion polymerase and primer pairs 84/85 (5′*GRE3*_AGS7), 86/87 (3′*GRE3*_AGS7), 84/109 (5′*GRE3*_AGS8) and 87/110 (3′*GRE3*_AGS8), generating PCR products with 50 bp homology to the flanks of *Sfa*AI/*Sgs*I cleaved plasmid AGS7 or *Sgs*I/*Ehe*I cleaved plasmids pAGS8B and pAGS8HB. For integration of pAGS8 into the *VAC17/MRC1* intergenic region, 400 bp of the 5′ upstream region and the 3′ downstream intergenic region between *VAC17* and *MRC1* were amplified from genomic CEN.PK 113-7D DNA using Phusion polymerase and primer pairs 88/89 (5′VAC17_AGS8) and *MRC1*_f/*MRC1*_r (3′*MRC1*_AGS8) generating PCR products with 50 bp homology to the flanks *Sfa*AI/*Sgs*I cleaved plasmid pAGS8. For integration of pAGS7, pAGS8 and pAGS8H the amdSYM marker was included and to enable marker recycling of amdSYM a 40 bp direct repeat with homology to the upstream region of amdSYM was included in the primers 86_3′*GRE3*_f_AGS7 and 3′*MRC1*_r_AGS8 (Solis-Escalante et al. 2013). A schematic picture of the integration of pAGS8B and pAGS8HB in the *GRE3* locus using CRISPR–Cas9 is shown in Additional file 2: Figure S1.

### Yeast strain engineering

The strains used and generated in this study are listed in Table 2. The upper part of the Weimberg pathway containing *xylB* and *xylC* (pAGS7) was integrated into the *GRE3* locus of CEN.PK 113-7D while the lower part of the Weimberg pathway containing *xylD*, *xylX* and *xylA* (pAGS8) was integrated into the *VAC17/MRC1* intergenic region using nested homologous recombination. For pAGS7 integration the strain was transformed with *Sfa*AI/*Sgs*I-digested pAGS7 and the targeting fragments for *GRE3*_AGS7 described above. pAGS8 or pAGS8H were integrated by transforming the strain with *Sfa*AI/*Sgs*I digested pAGS8/8H and the targeting fragments for VAC17/MRC1_AGS8 described above. Transformants were selected on SM-Ac (containing acetamide as the sole nitrogen source) and correct integration was validated with primer pairs 5/87 for pAGS7 and with 5/MRC1_r and 88/158 for pAGS8/pAGS8H. The amdSYM marker was recycled by growing the cells overnight in liquid YPD and plating a small amount of cells on SM-FAc where only cells that had lost the amdSYM marker should be able to survive (Solis-Escalante et al. 2013). Removal of the amdSYM marker was confirmed by PCR with primer pairs 155/87 for pAGS7 and 222/*MRC1*_r for pAGS8/pAGS8H. The resulting strains were named TMB4511 (pAGS7), TMB4515 (pAGS8), TMB4520 (pAGS7/8) and TMB4526 (pAGS7/8H).

**Table 2:** Strains used in the present study

Strain name	Relevant genotype	Encoded enzymes introduced into yeast	References
CEN.PK 113-7D	*MATa URA3 HIS3 LEU2 TRP1 MAL2*-*8*^*C*^ *SUC2*	–	Entian and Kötter (2007)
TMB4545	*VAC17/MRC1::TDH3p*-*GAL2*^*N376F*^-*CYC1t*-*FBA1p*-*TAL*-*PDC1t*-*TPI1p*-*TKL*-*CPS1t*, *X*-*2::XYL*-*XDH*-*XKS1; XI*-*5::XYL*-*XDH*-*XKS1, XII*-*4::XYL*-*XDH*-*XKS1*	XR, XDH (with an optimized PPP)	Unpublished
TMB4511	CEN.PK 113-7D, *Δgre3*::AGS7	XylB, XylC	This study
TMB4512	CEN.PK 113-7D, *Δgre3::xylB*	XylB	This study
TMB4515	CEN.PK 113-7D, vac17mrc1::AGS8	XylD, XylX, XylA	This study
TMB4520	CEN.PK 113-7D, *Δgre3*::AGS7, vac17mrc1::AGS8	XylB, XylC, XylD, XylX, XylA	This study
TMB4526	CEN.PK 113-7D, *Δgre3*::AGS7, vac17mrc1::AGS8H	XylB, XylC, XAD_Hv, XylX, XylA	This study
TMB4530	CEN.PK 113-7D, Δ*gre3*::AGS8B	XylB, XylD, XylX, XylA	This study
TMB4531	CEN.PK 113-7D, Δ*gre3*::AGS8HB	XylB, XAD_Hv, XylX, XylA	This study
TMB4569	CEN.PK 113-7D, Δ*gre3*::AGS8HB, Δ*xylD::xylD_Bc*	XylB, XylD_Bc, XylX, XylA	This study
TMB4570	CEN.PK 113-7D, Δ*gre3*::AGS8HB, Δ*xylD::yjhG_Ec*	XylB, YjhG_Ec, XylX, XylA	This study
TMB4571	CEN.PK 113-7D, Δ*gre3*::AGS8HB, Δ*xylD::xylD_El*	XylB, XylD_El, XylX, XylA	This study

XR: d-xylose reductase, XDH: xylitol dehydrogenase, PPP: pentose phosphate pathway, XylB: d-xylose dehydrogenase, XylC: Xylonolactonas, XylD: d-xylonate dehydratase, XylX: 2-keto-3-deoxy-d-xylonate dehydratase, XylA: α-ketoglutarate semialdehyde dehydrogenase, Hv: *Haloferax volcanii*, Bc: *Burkholderia cenocepacia*, Ec: *Escherichia coli*, El: Ellin329 isolate

In the second generation of strains, *xylC* was eliminated to allow a more gradual formation of d-xylonate and avoid the drastic drop in intracellular pH as previously reported (Nygård et al. 2014). First, a control strain containing only *xylB* was constructed. *TEFp*-*xylB*-*TEFt* was amplified from pAGS7 with primers 308/309 containing 50 bp flank to the *GRE3* locus and transformed together with gRNA.*GRE3*-natMX (LWA25) into CEN.PK 113-7D already containing Cas9-kanMX. Transformants were selected on YPD supplemented with geneticin and nourseothricin and validated by amplifying parts of the integrated cassette by PCR using primers 65/225. The resulting strain was named TMB4512. In a similar approach the constructed Weimberg genes present in pAGS8B and pAGS8HB was integrated into the *GRE3* ORF of *S. cerevisiae* CEN.PK 113-7D through homologous recombination using the CRISPR–Cas9 system and a gRNA that target the *GRE3* ORF as described above and in Additional file 2: Figure S1 (Stovicek et al. 2015). The yeast already expressing Cas9-kanMX was transformed with *Asc*I and *Ehe*I digested pAGS8B and pAGS8HB plasmids, the targeting fragments for *GRE3* and gRNA.*GRE3*-natMX (LWA25). Transformants were selected on YPD supplemented with geneticin and nourseothricin and validated by amplifying parts of the integrated cassette by PCR using primer pair 84/66. The resulting strains were named TMB4530 (pAGS8B) and TMB4531 (pAGS8HB). The strain TMB4530 containing pAGS8B (*xylB, xylD, xylX* and *xylA from C. crescentus*) was further engineered using CRISPR–Cas9 in order to replace the d-xylonate dehydratase from *C. crescentus* with three alternative putative *xylD* genes from *B. cenocepacia* (*xylD_Bc*), *E. coli* (*yjhG_Ec*) and *Ellin329* bacterium (*xylD_El*) resulting in three new strains named TMB4569, TMB4570 and TMB4571, respectively. These three strains were constructed by transforming TMB4530 containing Cas9-kanMX with either *Bam*HI and *Asc*I digested pUC57-xylD_Bc, pUC57-yjhG_Ec or pUC57-xylD_El together with the episomal plasmid gRNA.xylD-natMX (pLWA36). All the four *xylD* homologs were designed with the same promoter–terminator pairs (from *GPM1*) to allow homologous recombination and replacement of the *xylD* as shown in the schematic picture presented in Additional file 2: Figure S1 panel B. Transformants were selected on YPD supplemented with geneticin and nourseothricin and validated by amplifying the specific *xylD* homolog using primer pairs 269/270 (*xylD_El*), 271/272 (*yjhG_Ec*) and 273/274 (*xylD_Bc*).

### Reverse transcriptase-PCR experiments

Strains were grown in YPD to exponential phase and cells were lysed using a bead beater (Precellys 24, Bertin Technologies, France) connected to a cooling device (Cryolys, Bertin Technologies, France) according to the supplier’s instructions. RNA was extracted from the cell lysate using RNeasy Mini Kit (Qiagen, 74104) according to supplier’s instructions. DNA was degraded from the RNA samples using DNA free kit from Ambion (AM1906, Life Technologies, California, USA) and incubation for 1 h at 37 °C. Complete removal of DNA was confirmed by PCR directly on the RNA samples using primer pair 232/233 that amplify the constitutively expressed gene *PFY1* encoding Profilin. RNA concentration was determined using Biodrop DUO (Biodrop Ltd, Cambridge, England). 200 ng DNA-free RNA was then used for cDNA synthesis with Superscript IV RT polymerase and oligo d(T)_20_ primer. 2.5 µM oligo d(T)_20_ was mixed with 0.5 mM dNTPs and 200 ng RNA. The primer-RNA mix was heated to 65 °C for 5 min. After cooling on ice SSIV buffer, 5 mM DTT, RNAseOUT and 200 U of Superscript IV RT polymerase were added to the RNA primer mix and incubated at 50 °C for 10 min, followed by enzyme heat inactivation for 10 min at 80 °C. The cDNA was used for RT-PCR to verify that the Weimberg genes were transcribed in *S. cerevisiae* strains TMB4530, TMB4531, TMB4569, TMB4570 and TMB4571. Specific primers to amplify *xylB*, *xylD*, *xylD_El, yjhG_Ec, xylD_Bc, xad_Hv*, *xylX* and *xylA* are listed in Additional file 1: Table S1.

### In vitro enzymatic activity measurements

Overnight cultures were inoculated in 250 mL shake flasks containing 25 mL YNB supplemented with d-glucose (20 g L^−1^) at a starting optical density of 0.25 at 620 nm (Ultrospec 2100 Pro spectrophotometer, Amersham Biosciences Corp., USA) and grown at 30 °C and 180 rpm. The cells were harvested at late exponential phase and the total protein was extracted using Y-PER extraction solution (Pierce, Illinois, USA) according to manufacturer’s instructions. Total protein concentration was determined using Micro BCA™ Protein Assay (ThermoFisher Scientific, Massachusetts, USA) and bovine albumin standard (ThermoFisher Scientific, Massachusetts, USA) based on the assay developed by (Bradford 1976). All enzyme activities were assayed by following the rate of NAD^+^ reduction by measuring the optical density at 340 nm (OD_340 nm_, Ultrospec 2100 Pro spectrophotometer, Amersham Biosciences Corp., USA). The measurements were performed in duplicates at 30 °C on a Multiskan Ascent reader (Thermo Electro Corporation, Finland) using 96-well plates and a final volume of 200 µL. Before adding the substrate (d-xylose or d-xylonate) to the reactions, the background formation of NADH was monitored and subtracted, if present. The standard assay mixture for all the enzymatic reactions contained 100 mM Tris–HCl pH 8.0, 2 mM MgCl_2_ and 2 mM NAD^+^. A concentration of 100 mM of d-xylose was used as substrate for d-xylose dehydrogenase XylB (Toivari et al. 2012). The overall combined activity of the lower part of the pathway including XylD, XylX and XylA was assayed by following the formation of NADH using 80 mM d-xylonate as substrate (Meijnen et al. 2009). The d-xylonate dehydratase activity was also assayed using the thiobarbituric assay (TBA) method as described by Buchanan et al. (1999) and Salusjärvi et al. (2017). 10 µL of protein extract was mixed with 90 µL 10 mM d-xylonic acid in 10 mM MgCl_2_ and 50 mM Tris–HCl (pH 8.5) and incubated at 30 °C for 10 min. After the incubation, 10 µL 12% (w/v) trichloroacetic acid was added and precipitated proteins were removed by centrifugation. 50 µL of the supernatant was oxidized by addition of 125 µL 25 mM periodic acid/0.25 M H_2_SO_4_ and incubated at room temperature for 20 min. To terminate oxidation, 250 µL 2% (w/v) sodium (meta) arsenite in 0.5 M HCl was added. 1 mL 0.3% (w/v) thiobarbituric acid (TBA) was added and the chromophore developed for 10 min at 100 °C. To intensify the color, 100 µL of this sample was taken and added to 100 µL DMSO in a microtiter plate. The absorbance was read at 550 nm.

### Dot plating

A single-colony of each strain was grown in 5 mL of YNB supplemented with d-glucose in a 50 mL conical tube (Sarstedt AG & Co., Nümbrecht, Germany) and incubated at 30 °C and 180 rpm. At the end of the exponential growth phase, the cultivations were stopped and the amount of cells was normalized by dilution with 0.9% NaCl to an OD_620_ of 2.5, which corresponds to approximately 10^7^ colony forming units (CFU) per mL. Serial dilutions of each strain were done under sterile conditions with 0.9% NaCl to obtain a coverage from 10^5^ to 10^8^ CFU mL^−1^. 5 µL of each dilution were carefully dot plated in YNB-agar plates supplemented with d-glucose (5 g L^−1^), d-xylose (20 g L^−1^) or a mixture of d-glucose/d-xylose (5 and 20 g L^−1^, respectively) and incubated at 30 °C for 48 h.

### Aerobic cultivations in bioreactor

Bioreactor experiments were performed in lab-scale 3 L reactors Braun Biostat CT (B. Braun International, Germany) or Sartorius Biostat A+ (Sartorius, Germany) with an initial working volume of 1 L. Temperature was 30 °C, stirring 500 rpm using dual six-bladed Rushton turbines and sparging was 0.5 vvm air. As in the case of shake-flask cultivations, the medium also contained 6.7 g L^−1^ YNB with ammonium sulfate. For the batch experiments, the initial carbon source was 5 g L^−1^ d-glucose. When d-glucose was depleted, 20 g of d-xylose was added by injecting 50 mL of a 400 g L^−1^ d-xylose solution. pH was set to 5.5 and controlled by the addition of 5 M KOH. In the case of the fed-batch experiments, the initial substrate increased to 10 g L^−1^ d-glucose. d-Xylose was subsequently added to a concentration of 10 g L^−1^, by pulsing 50 mL of a 200 g L^−1^ d-xylose solution. During fed-batch mode a 40 g L^−1^ d-glucose solution was fed at a rate of 0.2 g d-glucose h^−1^.

### Aerobic cultivations in shake-flasks

Prior to the aerobic cultivation, yeast pre-cultures were obtained by transferring a loop of cells from the glycerol stocks into 5.0 mL of YNB defined medium (6.7 g L^−1^ Yeast Nitrogen Base without amino acids (Becton, Dickinson and Company, USA) supplemented with 20 g L^−1^ d-glucose. The pH of the medium was adjusted to 6.0 by the addition of H_2_SO_4_ or KOH. Cells were grown in a 50 mL sterile conical tube and incubated at 30 °C and 180 rpm until the end of the exponential growth phase was reached. At that moment, cells corresponding to an OD_620 nm_ of 0.1 were transferred to 100 mL YNB d-glucose in 1000 mL baffled shake-flasks and incubated at 30 °C and 180 rpm. This intermediate step was used for cell proliferation in order to start the aerobic cultivations with high levels of biomass. At the end of the exponential phase, an amount of cells corresponding to an OD_620 nm_ of 14 was harvested, centrifuged (5 min, 4000 rpm) and washed once by resuspension in 0.9% NaCl. Aerobic cultivations were then conducted in 250 mL baffled shake-flasks containing 25 mL of YNB defined media buffered to pH 6.0 and supplemented with 5 g L^−1^ d-xylose. After the inoculum, the shake-flasks were incubated at 30 °C and 180 rpm for 100 h. OD_620 nm_ was monitored over time and samples were taken for metabolite analysis using HPLC (cf. below). The final pH was measured at the end of the cultivations.

### Metabolite analysis and cell dry weight determination

The measurement of the extracellular concentrations of d-xylose and d-xylonate was done by ultra-performance liquid chromatography (UPLC, Waters, Massachusetts, USA) through a novel method specifically developed for the analysis of d-xylonate (Almqvist et al. 2017). Calibration was made with a standard solution containing α-ketoglutarate, d-xylose, d-xylitol, d-xylonate and d-glucose. Prior to analysis, samples were centrifuged (2 min, 13,000 rpm), filtered (0.2 µm) and diluted with ultra-pure H_2_O if necessary. d-Xylose concentrations were also measured using a Waters HPLC system equipped with RI-detector (45 °C cell temperature). The column was a 300 × 7.8 mm, 9 μm Aminex HPX-87P column (BIO-RAD, Hercules, CA, USA). The column temperature was 85 °C, the mobile phase (0.6 mL min^−1^) consisted of ultra-pure water (18.2 MΩ) from an ELGA Purelab Flex unit (Veolia Water, Paris, France). Metabolites were analysed using a Waters HPLC system equipped with RI-detector (45 °C cell temperature). The column was a 300 × 7.8 mm, 9 μm Aminex HPX-87H column (BIO-RAD, Hercules, CA, USA). The column temperature was 60 °C, the mobile phase (0.6 mL min^−1^) consisted of 5 mM H_2_SO_4_ in ultra-pure water. Calibration was made with a standard solution containing α-ketoglutarate, d-xylose, d-xylitol, acetate, ethanol and glycerol. d-Xylonate concentrations were also measured using the hydroxamate method (Lien 1959) as described by Toivari et al. (2010). The d-xylonate concentration in the samples was determined by comparison with a standard curve using lithium xylonate from Sigma-Aldrich. The cell dry weight (CDW) was obtained from optical density (OD_620_) through a correlation curve. CDW was initially determined in triplicate by centrifuging samples at 13,000 rpm for 5 min. The cells were washed by resuspension in ultra-pure water followed by centrifugation. The washed cells were then resuspended in ultra-pure water and transferred to pre-dried and pre-weighed 5 mL glass test tubes. The test tubes were dried in a convection oven (Termaks, Norway) at 105 °C for 16–24 h and then placed in a desiccator until equilibration to room temperature. After this analysis, it was determined that an OD_620_ of 1.0 corresponded to a CDW of 0.40 g L^−1^.

### Primary nucleotide accession numbers

The primary nucleotide accession numbers of the genes of bacterial/archaeal origin heterologously expressed in *S. cerevisiae* were obtained from GenBank or the Integrative Microbial Genome database (IMG) and are listed in Table 3. These Weimberg pathway genes codon-optimized towards *S. cerevisiae* was deposited in GenBank with the accession numbers listed in Table 3. The percent sequence identity with the *C. crescentus* XylD is presented for the four XylD homologs used and was obtained by aligning the protein sequences using the multiple sequence alignments tool Clustal Omega developed at the European Bioinformatics Institute (EMBL-EBI) (McWilliam et al. 2013).

**Table 3:** Nucleotide accession numbers of the bacterial/archeal genes used in the study

Gene name	Organism	Accession number			% seq. identity with *Cc* XylD
		GenBank	IMG	GenBank codon-opt^b^	
d-Xylose dehydrogenase *xylB*	*C. crescentus*	YP_002516237.1		MG681087	
d-Xylonolactonase *xylC*	*C. crescentus*	YP_002516236.1		MG681088	
d-Xylonate dehydratase *xylD*	*C. crescentus*	YP_002516235		MG681089	100
2KD-dehydratase *xylX*	*C. crescentus*	YP_002516239.1		MG681090	
AKG semialdehyde dehydrogenase *xylA*	*C. crescentus*	YP_002516238.1		MG681091	
d-Xylonate dehydratase *yjhG*	*E. coli*	WP_000116326.1		MG681092	29.4
d-Xylonate dehydratase *xad*	*H. volcanii*	WP_004041116.1		MG681093	18.6
d-Xylonate dehydratase	Ellin329 bact^a^		2626539782	MG681094	73.9
Dihydroxy-acid dehydratase	*B. cenocepacia H111*		2533426636	MG681095	62.7

^a^Full name: Ellin329 bacterium JGI 000192CP-E08

^b^GenBank accession number for genes codon optimized for *Saccharomyces cerevisiae*

## Results

### Expression of the Weimberg pathway from *C. crescentus* for d-xylose assimilation in *S. cerevisiae*

**Fig. 2:**
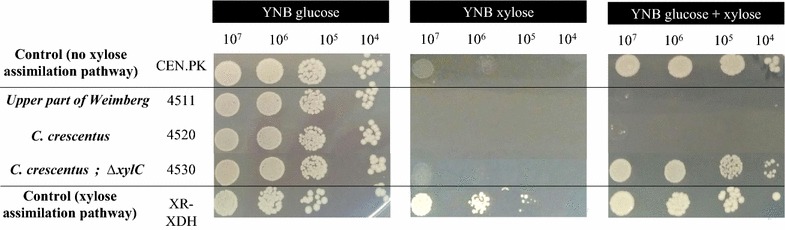
Evaluation of the growth capacity of yeast strains engineered with Weimberg pathway genes. The dot plating tests were done in defined agar-medium supplemented with d-glucose, d-xylose or a mixture of d-glucose and d-xylose. The background strain CEN.PK 113-7D was used as negative control for growth on d-xylose (first row). As a positive control for growth on d-xylose, a strain engineered with an optimized d-xylose oxido-reduction route (TMB4545) was used (last row)

Several different *S. cerevisiae* strains were constructed with varying combinations of the Weimberg pathway genes from *C. crescentus* integrated in the genome. In strain, TMB4511, only the first two genes, *xylB* and *xylC*, were integrated in the *S. cerevisiae* genome while in another strain, TMB4520, the entire Weimberg pathway genes were integrated (*xylB*, *xylC*, *xylD*, *xylX* and *xylA*). To prevent d-xylitol formation, *GRE3* encoding a native aldose reductase was deleted in all the *S. cerevisiae* strains carrying the Weimberg pathway. The growth capacity of the strains was evaluated by dot plating on media containing d-glucose, d-xylose or a mixture of both carbon sources (Fig. 2). When d-glucose was the sole carbon source, all strains grew, which was expected since the d-glucose metabolism is not directly connected with the d-xylose assimilation through the Weimberg pathway. However, when d-glucose was used in combination with d-xylose as carbon source, growth of strains carrying part or all Weimberg pathway genes (TMB4511 and TMB4520, respectively) was inhibited, indicating that d-xylose conversion, either in part or fully, through the Weimberg pathway was inhibitory to *S. cerevisiae*. This is in line with previous published results by Nygård and coworkers in 2014 who showed that the expression of the d-xylono-γ-lactone lactonase encoding gene *xylC* led to a rapid conversion of d-xylono-γ-lactone to d-xylonate, decreasing the intracellular pH and, consequently, initiating cell death. A more gradual d-xylonate formation was obtained by solely expressing the d-xylose dehydrogenase encoding gene *xylB* since the conversion of d-xylono-γ-lactone into d-xylonate also occurs spontaneously at neutral pH (Buchert and Viikari 1988; Nygård et al. 2014). In the present study, a strain was therefore constructed with the full Weimberg pathway from *C. crescentus* except for *xylC* integrated in the genome (Additional file 2: Figure S1). In this way, it was possible to evaluate whether the absence of the d-xylono-γ-lactone lactonase activity would also be beneficial when the complete Weimberg pathway was expressed in *S. cerevisiae*. The resulting strain, TMB4530, was indeed able to grow on d-glucose also in the presence of d-xylose in contrast to the strains containing *xylC* (TMB4511, TMB4520). However, TMB4530 was still not able to grow on d-xylose as the sole carbon source (Fig. 2).

### Expression of the Weimberg pathway genes from *C. crescentus* in *S. cerevisiae* enables bioconversion of d-xylose to d-xylonate via d-xylose dehydrogenase

**Fig. 3:**
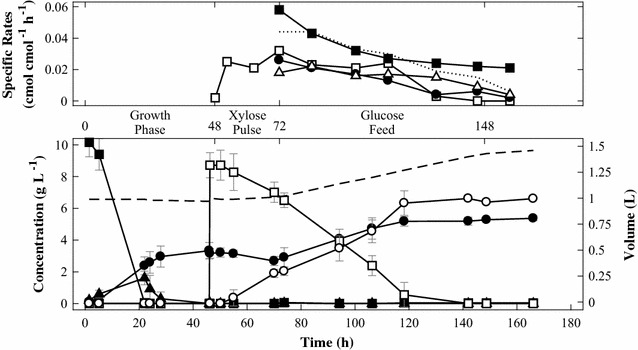
Fed-batch experiments with strain TMB4530 (*xylB*, *xylD*, *xylX*, *xylA*). d-Glucose (filled square), biomass (filled circle), ethanol (filled triangle), d-xylose (square), d-xylonate (circle), CO_2_ (triangle) and liquid volume (straight line). The dotted line represents the sum of specific productivity of biomass and CO_2_. All experiments were performed in biological duplicates and standard deviations are indicated with error bars

Strain TMB4530, containing *xylB*, *xylD*, *xylX* and *xylA*, was evaluated by bioreactor experiments in two steps: initial cell growth on d-glucose in a batch phase followed by a pulse addition of d-xylose to allow bioconversion at high cell density. In the first 24 h of the batch experiments, cells were propagated in YNB supplemented with d-glucose. After depletion of the d-glucose and the formed ethanol and acetate, the bioconversion phase was initiated by the addition of d-xylose. During this period, the specific d-xylose uptake rate (0.027 ± 0.002 cmol d-xylose cmol biomass^−1^ h^−1^) matched the d-xylonate production rate (0.024 ± 0.001 cmol d-xylonate cmol biomass^−1^ h^−1^) (data not shown). Cell growth could not be sustained by d-xylose during the bioconversion phase; instead biomass was estimated to decrease at a rate of 0.006 ± 0.001 h^−1^. In order to maintain cell viability, a fed-batch experiment was designed where d-glucose was fed during the bioconversion phase. The fed-batch experiment consisted of three phases (Fig. 3): initial growth phase on d-glucose followed by a pulse of d-xylose, where non-growing cells assimilated the pentose for 24 h. Finally, the third phase consisted of a prolonged fed-batch phase where d-glucose was fed at a slow rate of 0.2 g h^−1^. This set-up allowed the comparison of the d-xylose uptake efficiency by both growing and non-growing cells in the same experiment. The measurements showed that d-xylose uptake rate was not affected by d-glucose feeding. In the growth phase, d-glucose depletion was reached with TMB4530 after approximately 20 h of cultivation. After 46 h, a pulse of d-xylose was added and the pentose was taken up at a specific rate of 0.025 ± 0.004 cmol d-xylose cmol biomass^−1^ h^−1^ independent of the d-xylose concentration (Fig. 3). The assimilated d-xylose was converted to d-xylonate at a conversion rate of 0.020 ± 0.005 cmol biomass^−1^ h^−1^, reaching full conversion after 140 h. No other products could be detected from d-xylose.

### Expression of *xylD* from *H. volcanii* does not convey d-xylonate conversion

**Fig. 4:**
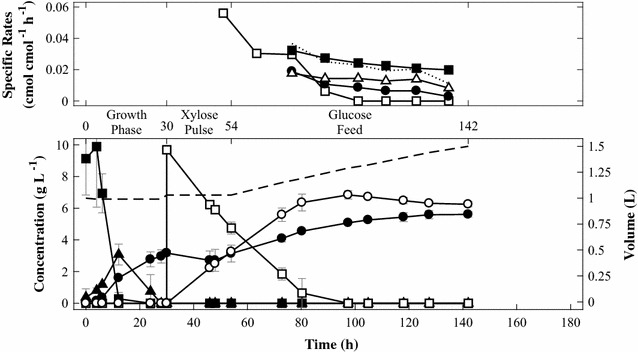
Fed-batch experiments with strain TMB4531 (*xylB*, *xad_Hv*, *xylX*, *xylA*). d-Glucose (filled square), biomass (filled circle), ethanol (filled triangle), d-xylose (square), d-xylonate (circle), CO_2_ (triangle) and liquid volume (straight line). The dotted line represents the sum of specific productivity of biomass and CO_2_. All experiments were performed in biological duplicates and standard deviations are indicated with error bars

Heterologous expression of the *C. crescentus* Weimberg pathway in *S. cerevisiae* enables bioconversion of d-xylose to d-xylonate, however, the d-xylonate is apparently not converted further as no downstream products can be detected. The dehydration reaction converting d-xylonate to 2-keto-3-deoxy-d-xylonate is catalysed by a dehydratase (XylD) belonging to the IlvD/EDD protein family that requires an iron–sulfur cluster (Fe–S) for its catalytic activity. Iron–sulfur cluster containing proteins of bacterial origin are known to be challenging to express in eukaryotes due to differences in the complicated cell machinery responsible for the loading of the Fe–S cluster onto the apoprotein (Benisch and Boles 2014). As an alternative to iron–sulphur cluster requiring *Cc* XylD, there are d-xylonate dehydratases found in Archaea that belong to the enolase family and require metal ions (such as Mg^2+^) for its function rather than Fe–S clusters (Johnsen et al. 2009; Petsko and Ringe 2004; Faller et al. 1977). Therefore, the *C. crescentus xylD* in strain TMB4530 containing *xylB, xylD, xylX* and *xylA* was replaced with the counterpart from the halophilic archaeon *H. volcanii* (HVO_B0038A, xad_Hv), generating strain TMB4531. The same three phase-set up as previously described for TMB4530 was used to test d-xylose conversion with a low simultaneous d-glucose co-consumption. In the first batch phase (24 h) cells were propagated in YNB supplemented with d-glucose to high cell density and then the second phase was initiated after 30 h by adding a pulse of d-xylose to allow bioconversion by the non-growing cells produced in the first phase. The third phase was a prolonged fed-batch phase where d-glucose was added at a slow rate of 0.2 g h^−1^ to maintain cell viability during the d-xylose bioconversion. TMB4531 reached a maximum uptake rate (0.06 cmol d-xylose cmol biomass^−1^ h^−1^) when the highest concentration of d-xylose was present, declining after that time-point. During the initial part of the fed-batch phase, the specific biomass and CO_2_ productivity were identical to TMB4530. In contrast, the carbon flux appeared to be shifted towards CO_2_ formation at later stages (Figs. 3, 4). The decrease in biomass yield correlated well with the d-xylonate concentrations, suggesting increased maintenance requirements due to the need of secreting the acid to maintain the intracellular pH, as previously suggested (Nygård et al. 2014). In both TMB4530 and TMB4531 the sum of biomass and CO_2_ production rates matched the d-glucose uptake rate (dotted lines in Figs. 3, 4), which suggested that d-glucose was converted into biomass and CO_2_ while d-xylose was quantitatively converted into d-xylonate (note that the concentrations decreased due to dilution during the fed-batch).

The increased d-xylose bioconversion rate in TMB4531 compared to TMB4530 could indicate that *H. volcanii* XylD (*Hv* XAD) was able to convert some of the d-xylonate to 2-keto-3-deoxy-d-xylonate, thereby increasing the intracellular pH and the fitness of the cells. 2-keto-3-deoxy-d-xylonate was not included in the standard solution used in the UHPLC and HPLC analyses since it is not commercially available. But as shown in Additional file 3: Figure S2, no unidentified peaks were seen in the chromatograms from the bioreactor experiments. Proteomics analysis of TMB4531 in a parallel study indicated that *Hv* XAD was not produced in TMB4531, while *Cc* XylD was present in TMB4530 (data not shown). Synthesis of the Fe–S *Cc* XylD in TMB4530 might increase the metabolic burden in this strain, which would explain the lower d-xylose bioconversion rate in TMB4530 in comparison to TMB4531 that lacks the Fe–S XylD protein.

### Evaluation of three XylD homologs from the IlvD/EDD family to improve d-xylonate conversion

Although *Cc* XylD was produced in TMB4530, the functionality of this protein remained unclear. In an attempt to improve d-xylonate conversion, we evaluated three XylD homologs of the IlvD/EDD family originating from *B. cenocepacia*, *E. coli* and an unknown organism denoted as Ellin329 bacterium, for their functionality in yeast by integration into TMB4530 replacing *xylD*, and resulting in strains TMB4569, TMB4570 and TMB4571, respectively (Additional file 2: Figure S1). These XylD homologs were chosen based on the difference in protein sequence identity as compared to *C. crescentus* XylD (Table 3) and the fact that they had previously not been characterized in *S. cerevisiae*.

**Fig. 5:**
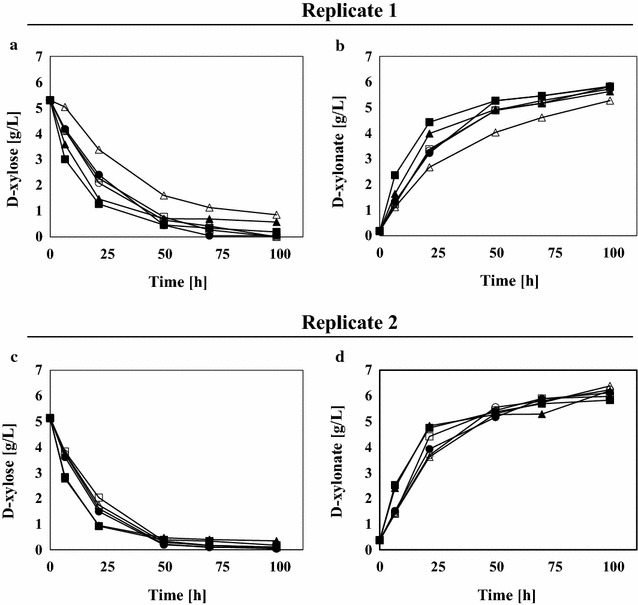
Aerobic bioconversion of d-xylose into d-xylonate in recombinant *Saccharomyces cerevisiae* strains. TMB4511 (*xylB*, *xylC*: filled square), TMB4512 (*xylB*: filled triangle), TMB4530 (*xylB*, *xylD*, *xylX*, *xylA*: filled circle), TMB4569 (*xylB*, *xylD_Bc*, *xylX*, *xylA*: square), TMB4570 (*xylB*, *yjhG_Ec*, *xylX*, *xylA*: triangle), TMB4571 (*xylB*, *xylD_El*, *xylX*, *xylA*: circle). All experiments were performed in biological duplicates. The d-xylose consumption and d-xylonate formation in replicate 1 is shown in the upper **a** and **b**, respectively. The lower **c** and **d** show d-xylose consumption and d-xylonate formation in replicate 2, respectively

Initial evaluation showed that all strains grew well on minimal medium supplemented with d-glucose, but no growth could be observed when d-xylose was used as the sole carbon source (data not shown). Subsequently, the biocatalytic capacities of the strains to convert d-xylose into d-xylonate and downstream products were assessed. For these tests, cells were first grown to exponential phase in medium containing d-glucose. After harvest, about 5 g L^−1^ biomass was transferred to a mineral medium supplemented with a small amount of d-xylose (5 g L^−1^) and aerobic cultivations were initiated in shake flasks (Fig. 5). For these experiments two control strains containing only part of the pathway were also evaluated under the same conditions namely TMB4511 containing *xylB* and *xylC* and TMB4512 containing only *xylB*. The results showed that both control strains TMB4511 and TMB4512 (i.e. the strain producing both XylB and XylC and the strain producing XylB, respectively) converted d-xylose to d-xylono-γ-lactone/d-xylonate at higher rates than all the strains containing genes from the lower pathway. This suggested that the lower part of the pathway put some metabolic burden on these strains since no downstream products from d-xylonate could be detected in the current set up. In strain TMB4511, rapid d-xylonate production was expected since the strain can enzymatically convert d-xylono-γ-lactone to d-xylonate; however this was also observed for the control TMB4512 that lacks XylC and has to rely on the spontaneous opening of the lactone ring to form d-xylonate. This can be explained by the analytical method used, called the hydroxamate method that in the current setup does not distinguish between the lactone and the acid form since all the d-xylonate is converted to d-xylono-γ-lactone and then measured colorimetrically.

### Investigation of non-functional steps for yeast strains carrying the whole Weimberg pathway

**Fig. 6:**
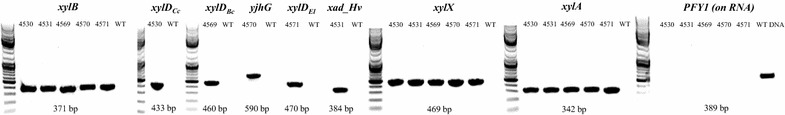
Reverse-transcriptase PCR analysis to verify gene expression of Weimberg pathway genes. RNA from five different strains exponentially growing on d-glucose was purified and used for cDNA synthesis. Reverse-transcriptase PCR was performed on the cDNA using primers specific for each Weimberg pathway gene. Amplification of *xylB*, *xylA* and *xylX* was carried out in all strains while different *xylD*s was amplified in each individual strain as follows: *xylD* from *Caulobacter crescentus* in TMB4530, *xad* from *Haloferax volcanii* in TMB4531, *xylD* from *Burkholderia cenocepacia* in TMB4569, *yjhG* from *Escherichia coli* in TMB4570 and *xylD* from Ellin329 isolate in TMB4571. CEN.PK 113-7D (WT) was used as negative control. Before cDNA synthesis the RNA purity (i.e. removal of DNA) was examined using a control PCR that amplifies the constitutively expressed gene *PFY1* encoding Profilin

As d-xylonate was the main product in all *S. cerevisiae* strains carrying the Weimberg pathway, we verified that each strain expressed the correct heterologous genes. Reverse transcriptase PCR was carried out on cDNA derived from exponentially growing cells in d-glucose (d-xylose was not added since the expression in yeast is constitutive and not regulated by d-xylose as in *C. crescentus*). The results confirmed that all the engineered *S. cerevisiae* strains expressed *xylB*, *xylX* and *xylA* and each strain expressed one unique *xylD* homolog: TMB4530-*xylD* from *C. crescentus*, TMB4569-*xylD* from *B. cenocepacia*, TMB4570-*yjhG* from *E. coli*, TMB4571-*xylD* from Ellin329 isolate, but also TMB4531 carrying *xad* from *H. volcanii* (Fig. 6). Due to the absence of XylD protein in the strain carrying *H. volcanii*, the other strains were also tested and the presence of the corresponding proteins was confirmed (data not shown).

As a third step, the activity of the Weimberg enzymes was assayed. The d-xylose dehydrogenase (XylB) activity was determined enzymatically in cell extract by monitoring the NADH formation at 340 nm and the results showed an activity of 0.2–0.3 µmol min^−1^ mg^−1^ in the different strains (Table 4). The activity of the lower part of the pathway including d-xylonate dehydratase (XylD), 2-keto-3-deoxy-d-xylonate dehydratase (XylX) and the α-ketoglutarate semialdehyde dehydrogenase (XylA) was assessed through the combined activity of the enzymes by following the formation of NADH from d-xylonate. However, no enzymatic activity could be detected (data not shown). In order to validate both oxidation assays, protein extract from d-xylose-grown *C. crescentus* cells was used as positive control and it showed enzymatic activity both for XylB and for the three enzymes XylD/XylX/XylA using d-xylose and d-xylonate as substrate, respectively, which confirmed the robustness of the assays. d-Glucose-grown *C. crescentus* showed no activity of the tested enzymes (unpublished data). To specifically assay the d-xylonate dehydratase activity, the thiobarbituric acid (TBA) method was also used (Buchanan et al. 1999; Salusjärvi et al. 2017). No activity could be detected, indicating that none of the XylD homologous were active in vitro (data no shown). This is in line with the recent report from Salusjärvi et al. (2017) where no XylD activity was detected unless *FRA2* gene was deleted.

**Table 4:** Specific activity of d-xylose dehydrogenase, XylB, in different recombinant *S. cerevisiae* strains

Strain	Spec. activity (µmol min^−1^ mg^−1^)	STDEV
TMB4511	0.21	± 0.04
TMB4530	0.23	± 0.05
TMB4569	0.31	± 0.04
TMB4570	0.24	± 0.03
TMB4571	0.31	± 0.04
TMB4515	0.00	± 0.03

The enzymatic activity of XylB that catalyzes the conversion of d-xylose into d-xylono-γ-lactone was assayed in five strains including: TMB4511 (XylB, XylC), TMB4530 (XylB, *Cc* XylD, XylX, XylA), TMB4569 (XylB, *Bc* XylD, xylX, xylA), TMB4570 (XylB, *Ec* YjhG_Ec, XylX, XylA) and TMB4571 (XylB, El XylD_El, xylX, xylA). As a control strain, TMB4515 that contains only the lower part of the pathway including *Cc* XylD, XylX and XylA was used

These results suggest that although the genes encoding the Weimberg pathway could be expressed and transcribed in *S. cerevisiae*, the functionality of the three proteins converting d-xylonate into α-ketoglutarate remained unknown. If the d-xylonate is converted further it is below detection limit in our experimental setup. Proteomics data could confirm that all the bacterial Fe–S XylDs were present in the correct strain, yet the assembly of the Fe–S cluster onto the apoprotein could be the limiting factor for producing an active protein.

## Discussion

In the present study, the expression of the genes encoding for the entire Weimberg pathway in the eukaryote *S. cerevisiae* had the objective to provide an alternative entry point for d-xylose into the yeast central carbon metabolism with possibilities to produce new compounds that are intermediates or derivatives of the TCA cycle. The *C. crescentus* Weimberg pathway was chosen since the corresponding genes had already been integrated and proven to be functionally expressed in the bacteria *C. glutamicum* and *P. putida* (Meijnen et al. 2009; Radek et al. 2014). In *S. cerevisiae*, the upper part of the pathway including *xylB* and *xylC* from *C. crescentus* has been successfully expressed, enabling d-xylonate production from d-xylose (Nygård et al. 2014). In a recent study of the Dahms pathway in *S. cerevisiae* it was also shown that deletion of *FRA2* encoding a repressor of iron regulon transcription resulted in some activity of the d-xylonate dehydratase (Salusjärvi et al. 2017).

None of the recombinant strains designed in the present study could sustain growth on d-xylose alone and only d-xylonate formation was detected. Although all genes were transcribed, it remained unclear whether the corresponding proteins were catalytically active and functional in *S. cerevisiae* since only the activity of the first enzyme XylB could be verified. The combined assay of the three last enzymes (XylD, XylX, XylA) as well as the XylD (TBA) assay gave no activity in any of the recombinant *S. cerevisiae* strains. Due to the lack of products downstream of d-xylonate, it is likely that the dehydration of d-xylonate to 2-keto-3-deoxy-d-xylonate, facilitated by the iron–sulfur (Fe–S) cluster containing protein XylD, was a major bottleneck to attain a fully active Weimberg pathway. Other studies on introduction of the Weimberg pathway into *Pseudomonas* species and *C. glutamicum* also showed d-xylonate accumulation during cultivations, and its conversion into 2-keto-3-deoxy-d-xylonate occurred only when other carbon sources were depleted. Therefore, the reaction catalyzed by XylD was seemingly the rate-limiting step for an efficient d-xylose oxidation into α-ketoglutarate, also in these bacterial hosts (Meijnen et al. 2009; Radek et al. 2014). However, it cannot be ruled out that the second dehydratase in the Weimberg pathway, XylX, which catalyzes the dehydration of 2-keto-3-deoxy-d-xylonate to α-ketoglutarate semialdehyde may also not be functional. This enzyme is not a Fe–S cluster containing protein and protein BLAST analysis (NCBI: National Center for Biotechnology Information) reveals that the first 122 amino acids of the XylX protein (total length, 140 amino acids) contain a conserved domain from the fumarylacetoacetate (FAA) hydrolase family protein that is part of the MhpD superfamily of proteins. Further insights into the functionality of the Weimberg pathway could be revealed by analysis of its intracellular metabolites. However, this is currently hampered by the lack of commercial standards.

A recent study on in depth characterisation of *Cc* XylD heterologously expressed in *E. coli* led to the identification of three conserved cysteine residues that coordinate the Fe–S binding essential for catalytic activity of the protein (Andberg et al. 2016). Although the formation of Fe–S clusters occurs spontaneously in vitro, systems for Fe–S biogenesis are required in vivo (Roche et al. 2013). In eukaryotes, the assembly of iron–sulfur clusters is carried out by two complex machineries, one present in the mitochondria (ISC: mitochondrial iron–sulfur cluster) and one present in the cytosol (CIA: cytosolic iron–sulfur protein assembly) (Lill et al. 2015; Lill and Mühlenhoff 2008). These machineries assemble Fe–S clusters on scaffold proteins using iron and cysteine as the source of sulfur, and the Fe–S cluster is then transferred to apoproteins with the aid of specific chaperones (Lill and Mühlenhoff 2008). The chances of a correct formation of bacterial Fe–S cluster harboring proteins in *S. cerevisiae* remains unknown, since the biogenesis systems are significantly different in prokaryotes and eukaryotes (Kirby et al. 2016). However, examples of challenging functional expression of bacterial genes encoding Fe–S cluster harboring enzymes in eukaryotes have been reported. For instance, the prokaryotic 6-phosphogluconate dehydratase (PGDH) from *E. coli* is a Fe–S cluster-harboring enzyme, and despite a number of attempts to activate the Fe–S cluster machinery in yeast by increasing the intracellular iron availability, inducing iron-responsive genes or by localizing PGDH to the mitochondria, the activity and functionality of PGDH could not be improved (Benisch and Boles 2014). An insufficient loading of the Fe–S cluster onto the protein was hypothesized to result in a very low enzyme activity, since the Fe–S cluster machinery in *S. cerevisiae* failed to recognize it as an apoprotein (Benisch and Boles 2014). In a similar line, the activity of the bacterial enzyme IspG, whose activity is also dependent on the correct assembly of Fe–S clusters, was demonstrated to be the limiting step to obtain a functional heterologous pathway for the production of isoprenoids in *S. cerevisiae* (Carlsen et al. 2013; Kirby et al. 2016). In our case, all Fe–S XylD homologs were detected, indicating again that the translation and folding of the proteins may not be the problem, whereas the proper loading of the Fe–S cluster might be.

In an attempt to circumvent the Fe–S cluster problem, we searched for non Fe–S XylD homologs. In previous work focusing on enolase dehydratases, several six carbon dehydratases using d-mannonate or d-gluconate as substrate has been reported (Wichelecki et al. 2014) but no bacterial enzyme that can dehydrate d-xylonate has yet been found [reviewed by (Gerlt et al. 2012)]. Reported non Fe–S XylD come from extremophilic archaea such as the halophilic *H. volcanii* (Johnsen et al. 2009) and the thermophilic *Sulfolobus solfataricus* (Nunn et al. 2010). Based on the lower temperature optimum of *H. volcanii* (42 °C) it was chosen over the thermophilic *S. solfataricus* (75–85 °C) for this study. Thus, *xad* from *H. volcanii* (*xad_Hv*) was expressed in *S. cerevisiae* together with *xylB*, *xylX* and *xylA* from *C. crescentus* (strain TMB4531). The use of *Hv* XAD in TMB4531 increased the uptake and formation rates of d-xylose and d-xylonate, respectively, which could indicate some flux downstream of d-xylonate. Supporting this is the fact that the carbon recovery in the fed-batch experiment with d-xylose and d-glucose was 85% for TMB4531, while for TMB4530 harboring XylD from *C. crescentus* it was 100%. However, no further intermediates of the Weimberg pathway could be detected and no peptides corresponding to XAD could be found in TMB4531. Therefore, it is more likely that measurement error contributed to this result seen in the bioreactor experiment. Functional expression of genes originating from halophiles such as *H. volcanii* can be challenging since these organisms require 100–150 g L^−1^ salt to grow and maintain structural stability. The proteome of halophiles is usually acidic leading to protein denaturation when suspended in low salt (Oren 2008). Thus, the absence of XAD in *S. cerevisiae* strain TMB4531 could indicate that the protein folding of this halophilic protein was indeed problematic, and the increased d-xylose bioconversion rate observed in this strain in comparison to TMB4530 was probably caused by an increased metabolic burden in TMB4530 expressing and assembling the Fe–S cluster-containing XylD.

In conclusion, the dehydration reaction(s) appear to be major bottlenecks to establish an active Weimberg pathway in *S. cerevisiae*. Bacterial XylDs require assembly of Fe–S clusters for a functional active enzyme while archaeal XylDs of the enolase family originating from halophiles require high salt concentration for correct folding. At the time of writing, the hypothesis of a non- or poorly functional XylD was confirmed in a study where the Fe–S cluster metabolism of *S. cerevisiae* was altered by deletion of *FRA2* that regulates iron regulon transcription (Salusjärvi et al. 2017). This resulted in an increased activity of XylD from *C. crescentus* in yeast. Moreover, the XylD activity was improved when its expression was targeted to the mitochondrium, indicating that the cytosolic Fe–S cluster synthesis was inefficient in yeast (Salusjärvi et al. 2017). Further evaluation of this strategy as well as alternative d-xylonate dehydratases with different requirements and properties, is therefore expected to lead to significant improvements of Weimberg functionality in yeast. Similarly, the crystallization of *H. volcanii* XAD could open new routes for optimization of this protein functionality through protein engineering.

## Additional files

**Additional file 1: Table S1.** Primers used in the present study.

**Additional file 2: Figure S1.** Schematic representation of the integration of the Weimberg pathway encoding genes into *S. cerevisiae*. The CRISPR–Cas9 system and nested homologous recombination was used to integrate *xylB*, *xylX, xylA* together with *xylD* or *xad* from plasmid pAGS8B or pAGS8HB, respectively, replacing the *GRE3* ORF (panel A). This resulted in strain TMB4530 (*xylB*, *xylD*, *xylX* and *xylA*) and strain TMB4531 (*xylB*, *xad*, *xylX* and *xylA*). The 20 bp gRNA sequence used to target the Cas9 nuclease to the *GRE3* ORF is shown in bold. The CRISPR–Cas9 system was also used to replace *xylD* from *Caulobacter crescentus* in TMB4530 with three different homologs from *Burkholderia cenocepacia*, *Escherichia coli* and Ellin329 isolate generating strains TMB4569, TMB4570 and TMB4571, respectively (Panel B). The 20 bp gRNA sequence used to target the Cas9 nuclease to the *C. crescentus xylD* in TMB4530 is shown in bold.

**Additional file 3: Figure S2.** UHPLC and HPLC chromatograms of end point samples from the bioreactor experiments. Panel A and B show UHPLC chromatograms for strain TMB4530 (A) and TMB4531 (B) at the end of the fermentation experiment. No unidentified peaks could be found indicating that no detectable amounts of Weimberg intermediates were produced. Panel C and D show HPLC chromatograms for strain TMB4530 (C) and TMB4531 (D) from the same sample. One extra small peak can be seen (medium component), which was present at the start of fermentation thereby eliminating the possibility that this peak contains Weimberg intermediates.

## Abbreviations

AGE: agarose gel electrophoresis
AKG: α-ketoglutarate
Bc: *Burkholderia cenocepacia*
CDW: cell dry weight
CIA: cytosolic iron–sulfur protein assembly
Ec: *Escherichia coli*
El: Ellin329 isolate
HPLC: high-performance liquid chromatography
Hv: *Haloferax volcanii*
ISC: mitochondrial iron–sulfur cluster
LB: Luria–Bertani broth
NAD^+^/H: nicotinamide adenine dinucleotide
PCR: polymerase chain reaction
PGDH: 6-phosphogluconate
PPP: pentose phosphate pathway
SM: synthetic media
TCA cycle: tricarboxylic acid cycle
UHPLC: ultra-performance liquid chromatography
XDH: d-xylitol dehydrogenase
XI: d-xylose isomerase
XR: d-xylose reductase
YNB: yeast nitrogen base
YPD: yeast peptone dextrose media
Y-PER: yeast protein extraction reagent
