# Supplementary material for: Exploring d-xylose oxidation in Saccharomyces cerevisiae through the Weimberg pathway
Source: AMB Express. 2018 Mar 5;8:33. doi: 10.1186/s13568-018-0564-9 (PMC5838027; doi:10.1186/s13568-018-0564-9)
Supplement: Supplementary file 1 — Additional file 1: Table S1. Primers used in the present study. [file 13568_2018_564_MOESM1_ESM.docx]

| **Name** | Amplification target | Sequence (5’ to 3’)* |
| --- | --- | --- |
| 5_3'amdSYM | 3'amdSYM down | CCAGATGCGAAGTTAAGTGC |
| 84_5'GRE3_f | 5'GRE3 | TGATGAATTCGTAGACGCAG |
| 85_5'GRE3_r_AGS7 | 5'GRE3_AGS7 | agtaaaaaaggagtagaaacattttgaagctatactcttatcgggggcgcTCTTGAAGGATTGCACTGAC |
| 86_3'GRE3_f_AGS7 | 3'GRE3_AGS7 | gtaatatcatgcgtcaatcgtatgtgaatgctggtcgctatactggcgatcaaatttcaactgttatatagggaggcgccaacccttaatGCACTAAATGCCAACATCAG |
| 87_3'GRE3_r | 3'GRE3 | GCTTTGCTCTCTTGGTGTCT |
| 88_5'VAC17_f | 5'VAC17_f_AGS8 | ACGATCAGATACACATACGG |
| 89_5'VAC17_r | 5'Vac17_r_AGS8 | cttaaagtcatacattgcacgactacacgtgacccgggggcgcgCAAAAAAACTTGAAGATATTCTG |
| 3’MRC1_f | 3’MRC1_r_AGS8 | gcgtcaatcgtatgtgaatgctggtcgctatactggcgatttaatttttatttcattctggaactcttcgagttctttgTGTCTTATAAAATCCTATCATAACATGACTATG |
| 3’MRC1_r | 3’MRC1_r_AGS8 | CAAAAGCTTCTATCACATATATGGG |
| 208_MCS_Cassette_f | pUG-amdSYM | aggtcgacggcgcgcccccgggaggcgcCAACCCTTAATATAACTTCGTATAATGTATG |
| 209_MCS_Cassette_r | pUG-amdSYM | GAGAGCTCGCGATCGCCAGTATAGCGACCAGCATTC |
| LW102_gRNA GRE3_f_tail | gRNA backbone | ggcgcagatgacgagaagaaGTTTTAGAGCTAGAAATAGCAAG |
| LW103_gRNA SNR52p_r | gRNA | GATCATTTATCTTTCACTGC |
| LW109_5'GRE3_r_AGS8b | *GRE3* targeting fragment | gtaaaaaaggagtagaaacattttgaagctatactcttatcgggggcgcgTCTTGAAGGATTGCACTGAC |
| LW110_3'GRE3_f_AGS8b |  | gttttaatttttatttcattctggaactcttcgagttctttgtgggaggcGCACTAAATGCCAACATCAG |
| HvXAD_f | *xad_Hv* | GGTTGAAGAAGATGACTTGATACAAAAC |
| GPM1t_r | *GPM1t* | TACCGCATCAGGCGCCATATTCGAACTGCCCATTCAG |
| 155_TPI1t_f | *TPI1t* | cacatgggttaaTAATATAATTATATAAAAATATTATCTTCTTTTC |
| 158_GPM1p_r | *GPM1p* | ctttaccagtggccatTATTGTAATATGTGTGTTTGTTTG |
| 220_RT_xad_Hv_f | *xad_Hv* | GGTAGTGCAAAGAGATTGGC |
| 221_RT_xad_Hv_r |  | CCAACCCAATTGGTATGAGTG |
| 222_RT_xylA_f | *xylA* | TACATTGACATTGCCGCATC |
| 223_RT_xylA_r |  | ATGATAGTCAACTCCCGCAG |
| 224_RT_xylB_f | *xylB* | GACGTTTTGGTCAACAATGC |
| 225_RT_xylB_r |  | GCTGCTACTATCTGTGCTTC |
| 226_RT_xylX_f | *xylX* | CTACTGTGTGATGGTTCAGG |
| 227_RT_xylX_r |  | TAGCCTTGTCACAAGTTGTC |
| 228_RT_xylD_f | xylD | AAGAGGTACTTATCACAGCC |
| 229_RT_xylD_r |  | GTACTGCAGGAATACCGTCT |
| 230_RT_xylC_f | *xylC* | ATTGTGGTTTGGCACTATGC |
| 231_RT_xylC_r |  | GTCCGACAAACCTTTTCTGG |
| 232_RT_PFY1_f | *PFY1* | AGCTCAGTTGACCCTTTCTC |
| 233_RT_PFY1_r |  | ACTTTTGGCCTTGAATATGC |
| 241_RT_GRE3_f | *GRE3* | CATCACCGAAGCACATGTACC |
| 242_RT_GRE3_r |  | CTCTGAGTTGCCCATCTAAGC |
| 263_xylD_gRNA | gRNA backbone | gctttaggtttgtccctgacGTTTTAGAGCTAGAAATAGCAAG |
| 269_xylD_El_f | xylD_El | GGTAGATTGGATGGTAGTGC |
| 270_xylD_El_r |  | AGATGGAGAATCAGAAGTACC |
| 271_yjhG_Ec_f | yjhG_Ec | TCTCCACAACAAGCTAAAGC |
| 272_yjhG_Ec_r |  | GTATCATCAGGCAATTCTGG |
| 273_xylD_Bc_f | xylD_Bc | TCCGGTAATTTCTTCGATTCC |
| 274_xylD_Bc_r |  | CCAATTCATCTTCATCGACC |

**Table S1 Primers used in the present study**

* Uppercase letters indicate the primer annealing region while lowercase contains extra features such as enzyme cleavage sites or homology regions. Bold lowercase letters indicate a direct repeat region in the primer.

**Figure S1 Schematic representation of the integration of the Weimberg pathway encoding genes into *S. cerevisiae*.** The CRISPR-Cas9 system and nested homologous recombination was used to integrate *xylB*, *xylX, xylA* together with *xylD* or *xad* from plasmid pAGS8B or pAGS8HB, respectively, replacing the *GRE3* ORF (panel A). This resulted in strain TMB4530 (*xylB*, *xylD*, *xylX* and *xylA*) and strain TMB4531 (*xylB*, *xad*, *xylX* and *xylA*). The 20 bp gRNA sequence used to target the Cas9 nuclease to the *GRE3* ORF is shown in bold. The CRISPR-Cas9 system was also used to replace *xylD* from *Caulobacter crescentus* in TMB4530 with three different homologs from *Burkholderia cenocepacia*, *Escherichia coli* and Ellin329 isolate generating strains TMB4569, TMB4570 and TMB4571, respectively (Panel B). The 20 bp gRNA sequence used to target the Cas9 nuclease to the *C. crescentus xylD* in TMB4530 is shown in bold.

**Figure S2 UHPLC and HPLC chromatograms of end point samples from the bioreactor experiments**

Panel A and B show UHPLC chromatograms for strain TMB4530 (A) and TMB4531 (B) at the end of the fermentation experiment. No unidentified peaks could be found indicating that no detectable amounts of Weimberg intermediates were produced. Panel C and D show HPLC chromatograms for strain TMB4530 (C) and TMB4531 (D) from the same sample. One extra small peak can be seen (medium component), which was present at the start of fermentation thereby eliminating the possibility that this peak contains Weimberg intermediates.
